# 大型仪器支撑下的样品前处理教学改革实践——以磁性共价有机骨架材料富集海水中有机紫外防晒剂为例

**DOI:** 10.3724/SP.J.1123.2025.08010

**Published:** 2026-06-08

**Authors:** Shuang LI, Yiling ZHU, Zhen REN, Dangdang GAO, Helin GU, Junlang LAO

**Affiliations:** 青岛理工大学环境与市政工程学院，山东 青岛 266520; School of Environmental and Municipal Engineering，Qingdao University of Technology，Qingdao 266520，China

**Keywords:** 综合化学实验, 液相色谱-串联质谱法, 磁固相萃取, 教学改革, 环境化学, integrated chemistry experiment, liquid chromatography-tandem mass spectrometry （LC-MS/MS）, magnetic solid-phase extraction （MSPE）, teaching reform, environmental chemistry

## Abstract

当前本科生环境化学实验教学中，样品前处理与大型仪器分析环节割裂，难以培养学生系统的分析能力；同时，实验内容仍以常规水质指标和重金属等传统污染物为主，缺乏针对新污染物的监测与治理技术训练，与国家战略需求存在显著差距。为此，本文以典型新污染物——药物及个人护理用品中的有机紫外防晒剂（OUVFs）为目标物，设计了一个融合材料合成、样品前处理与大型仪器分析于一体的综合性实验。实验采用加热回流法制备磁性共价有机骨架材料（MCOF），引导学生将其作为新型吸附剂应用于磁固相萃取（MSPE），利用其简便的磁分离性能在8 min内实现OUVFs的快速富集，结合超高效液相色谱-串联质谱法（UHPLC-MS/MS）完成海水样品的定量检测。该实验通过“课前预习-课中实验-课后讨论”三段式教学模块，重点强化学生在吸附剂合成与表征、富集参数优化及数据解析方面的自主探究能力。实践表明，该设计不仅使学生深入理解了复杂样品前处理过程及多因素协同作用机制，更显著提升了其在解决新污染物监测问题中的综合创新与实践能力。

## Abstract (en)

Current undergraduate environmental chemistry curricula often suffer from a disconnect between sample preparation and large-scale instrumental analysis， making it difficult to cultivate students’ systematic analytical capabilities. Furthermore， the experimental content predominantly focuses on conventional water quality parameters and traditional pollutants like heavy metals， lacking training in monitoring and remediation technologies for new pollutants， which creates a significant gap with national strategic needs. To address this issue， this study designed an integrated comprehensive experiment that combines material synthesis， sample pretreatment， and large-scale instrumental analysis， targeting typical new pollutants， such as organic ultraviolet filters （OUVFs） from pharmaceuticals and personal care products. A magnetic covalent organic framework （MCOF） was synthesized via a reflux heating method. Students were guided to apply it as the sorbent in magnetic solid-phase extraction （MSPE）. Owing to its facile magnetic separation， the rapid enrichment of OUVFs was achieved within 8 min， followed by quantitative determination of seawater samples using ultra-high performance liquid chromatography-tandem mass spectrometry （UHPLC-MS/MS）. Through a three-stage teaching module encompassing “pre-class preview， in-class experiment， and post-class discussion”， the experiment emphasizes enhancing students’ independent exploration abilities in adsorbent synthesis and characterization， optimization of enrichment parameters， and data analysis. Teaching practice has shown that this design not only enabled students to deeply understand the complex sample pretreatment process and the mechanism of multi-factor synergistic effects， but also significantly enhanced their comprehensive innovation and practical abilities in solving new pollutant monitoring problems.

随着国家“新污染物治理”战略的深入推进，环境监测领域对分析技术提出了“高通量、高灵敏度、绿色环保”的新要求^［1］^。在此背景下，环境化学实验教学改革必须紧跟国家战略需求，将学科前沿技术有机融入实践教学体系。基于成果导向教育（OBE）理念，以磁固相萃取（MSPE）技术为切入点开展实验教学创新，这不仅契合当前环境监测领域的技术发展趋势，更能有效培养学生的创新能力和实践技能。实验教学中，通过贯彻教育部《高等学校课程思政建设指导纲要》，充分发挥国产大型仪器的教学功能，将“重大科学仪器设备开发”国家专项的技术成果转化为优质教学资源^［2］^。这种综合型实验教学模式，既能提升学生的专业技术水平，又能培养其服务国家战略需求的使命担当，为培养新时代复合型环境专业人才提供有力支撑。

有机紫外防晒剂（OUVFs）作为典型的新污染物，其环境行为和生态风险正引发广泛关注^［3，4］^。这类化合物因其显著的强亲脂性和难降解性，已被认定为持久性有机污染物。通过防晒霜等个人护理用品的广泛使用，OUVFs经由日常洗浴、游泳等人类活动持续输入水环境并不断累积^［5，6］^。研究报道，部分滨海旅游区OUVFs的环境浓度已高达102.1 ng/L^［7］^。它们还表现出较强的生物毒性与内分泌干扰效应，已被列为《海洋生态环境保护“十四五”规划》重点监测对象^［8，9］^。超高效液相色谱-串联质谱法（UHPLC-MS/MS）是OUVFs检测的常用手段^［10］^，但受限于实际样品的复杂性和目标物的超低浓度，高效的样品前处理技术是不可或缺的环节。MSPE技术凭借其独特的磁性分离优势，结合功能化吸附剂的选择性富集能力，为满足环境监测领域分析需求提供创新性技术方案^［11-13］^。

在MSPE技术中，磁性吸附剂的选择至关重要。共价有机骨架材料（COFs）由C、H、O、N、B等轻元素通过共价键构建而成，具有规则的孔道结构和高比表面积^［14-16］^。基于此，本实验合成了磁性COF（MCOF）材料，实现OUVFs的选择性富集。首先，学生通过视频教学掌握MCOF的合成方法；随后在实验环节，通过优化样品前处理条件、建立UHPLC-MS/MS检测方法，初步了解海水中OUVFs的暴露水平，从而加深对环境污染现状的认知。通过对方法检出限、定量限、线性范围与富集因子等关键参数的测算，学生进一步理解了样品前处理技术在复杂环境样品中实现高灵敏分析的重要价值，全面提升其系统性实验技能与就业竞争力。

## 1 实验部分

### 1.1 仪器、试剂与材料

Triple Quad 3500型超高效液相色谱-串联质谱仪（美国AB Sciex公司）；Frontier型傅里叶变换红外光谱仪（FT-IR，美国ThermoFisher科技公司）；Sigma 300型扫描电子显微镜（SEM，德国ZEISS公司）；Millipore D-24UV超纯水机（美国Millipore公司）。

联苯胺（BD，纯度95%）和1，3，5-三（对甲酰基苯基）苯（TFPB，纯度99.5%）购自吉林中科研伸科技有限公司。Fe_3_O_4_（200 nm）和四氢呋喃（THF）购自上海麦克林生化科技有限公司；色谱级甲醇购自德国默克公司。2-氰基-3，3-二苯基丙烯酸-2-乙基己酯（OC）、对甲氧基肉桂酸辛酯（OMC）、水杨酸-2-乙基己酯（EHS）和1-（4-叔丁基苯基）-3-（4-甲氧基苯基）-1，3-丙二酮（AVO）均购自天津希恩思生化科技有限公司，其结构式如图1所示。

**图1:**
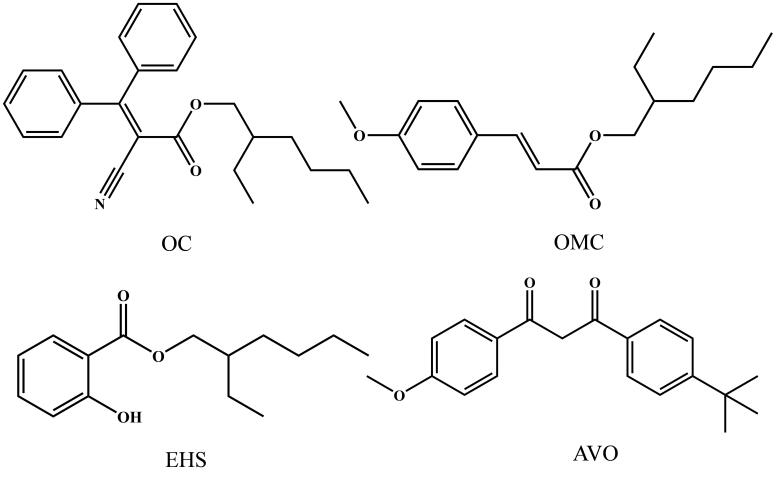
4种OUVFs的结构式

OUVFs混合标准储备液质量浓度为1 000 mg/L（溶剂为甲醇），置于4 ℃避光条件下保存。实际水样采集自青岛近岸海水，使用0.45 μm滤膜过滤后，储存于棕色玻璃瓶中以备使用。

### 1.2 吸附剂的教学合成过程（教师制备，视频演示）

采用加热回流法制备Fe_3_O_4_@TFPB-BD材料^［13］^，合成路线如图2所示。将直径为200 nm的Fe_3_O_4_颗粒（100 mg）与BD（36.8 mg）在20 mL THF中超声分散30 min，再机械搅拌30 min。然后，缓慢加入4 mL含有TFPB（78 mg）的THF溶液，在60 ℃下机械搅拌2 h，利用TFPB与氨基发生动态共价缩合反应，在磁核表面原位构筑具有高比表面积的COF壳层。反应结束后，用磁铁收集棕色磁性颗粒，并用甲醇洗涤4次，置于真空干燥箱中60 ℃下干燥。采用SEM和FT-IR对Fe_3_O_4_@TFPB-BD进行表征，揭示其形貌特征和化学键合成方式。本实验模块旨在引导学生理解共价有机骨架材料的合成机制及其微观、化学结构。

**图2:**
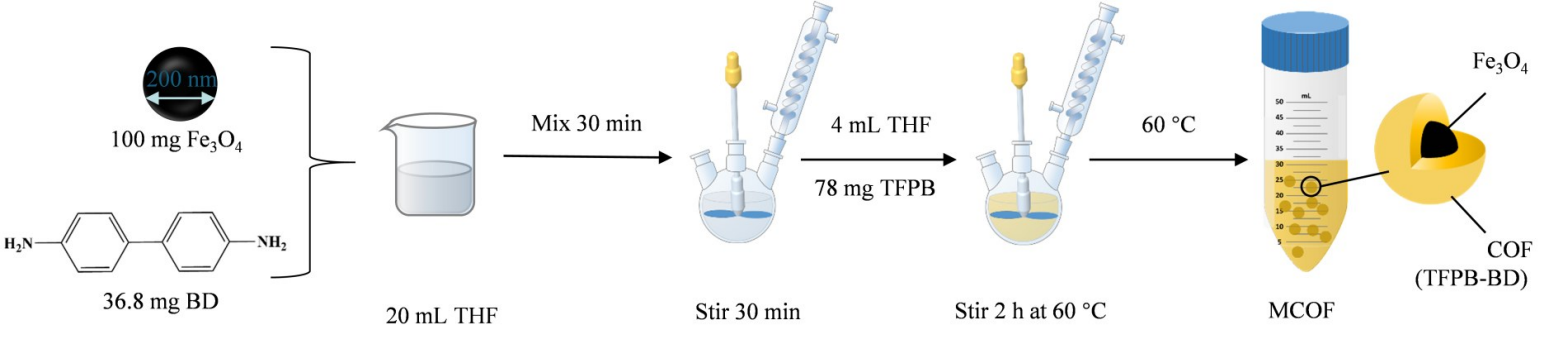
Fe_3_O_4_@TFPB-BD的合成路线图

### 1.3 磁固相萃取过程

将所合成的Fe_3_O_4_@TFPB-BD材料作为吸附剂，开展OUVFs的MSPE实验。操作流程如图3所示，称量14 mg Fe_3_O_4_@TFPB-BD，投入50 mL pH调节为8.0的海水样品中，涡旋振荡5 min。随后，利用外加磁场进行快速相分离，弃去上清液。向收集的材料中加入1 mL甲醇进行洗脱，涡旋3 min。磁分离后，将洗脱液经有机系滤膜过滤，待UHPLC-MS/MS定量检测。

**图3:**
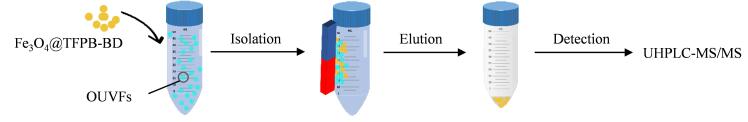
磁固相萃取流程图

### 1.4 UHPLC-MS/MS分析

采用ACQUITY UPLC BEH C18色谱柱（100 mm×2.1 mm， 1.7 μm），柱温40 ℃，进样量为5 μL，流速为0.3 mL/min。流动相由0.1%甲酸水溶液（A）和甲醇（B）组成。水相流动相（A）经0.22 μm水系滤膜过滤。采用梯度洗脱程序，具体如下：0~1.0 min， 60%A； 1.0~8.0 min， 60%A~1%A； 8.0~13.0 min， 1%A； 13.0~13.1 min， 1%A~60%A； 13.1~14.0 min， 60%A。

质谱采用电喷雾电离源（ESI）正离子模式，数据采集方式为多反应监测；离子源电压为5 500 V，离子源温度为450 ℃。4种OUVFs的质谱参数见表1。

**表1:** 4种OUVFs的理化性质及质谱参数

Analyte	*M*_r_	p*K*_a_	lg *K*_ow_	*t*_R_/min	Precursor ion （*m/z*）	Product ions （*m/z*）	Declustering potentials/V	Collision energies/eV
OC	361.5	7.5	6.4	8.7	362.1	250.1^*^， 231.9	45.4， 50.0	12.0， 29.0
OMC	290.4	7.5	5.0	9.0	291.3	179.0^*^， 161.0	45.8， 36.5	12.9， 26.4
EHS	250.3	8.1	4.4	9.2	251.1	120.9^*^， 138.9	33.3， 36.5	33.7， 12.6
AVO	310.4	9.7	4.3	8.9	311.0	161.0^*^， 135.1	52.9， 55.9	35.0， 30.7

* Quantitative ion.

## 2 结果与讨论

### 2.1 材料表征

如图4a所示，Fe_3_O_4_@TFPB-BD复合材料呈现直径约为200 nm的球状颗粒。通过FT-IR对其化学结构进一步表征（图4b），在1 298和1 443 cm^-1^处分别出现C-N和C=N的特征吸收峰，表明COF骨架的成功构建^［13］^。此外，在1 509和1 593 cm^-1^处出现的芳香碳骨架振动峰进一步佐证了COF的结构完整性。在580 cm^-1^处观察到Fe-O振动峰，证实了Fe_3_O_4_纳米颗粒已成功引入，从而验证Fe_3_O_4_@TFPB-BD复合材料的形成^［13］^。在本部分教学实践中，我们首先指导学生利用SEM和FT-IR对材料进行基础表征。在此基础上，我们进一步向学生阐明，完整的确证材料结构还需结合X射线衍射仪分析晶体构型、X射线光电子能谱探测表面元素化学状态等重要手段；并强调通过多种表征技术相互验证，构建可靠的材料结构体系，从而培养学生系统性的表征解析能力。

**图4:**
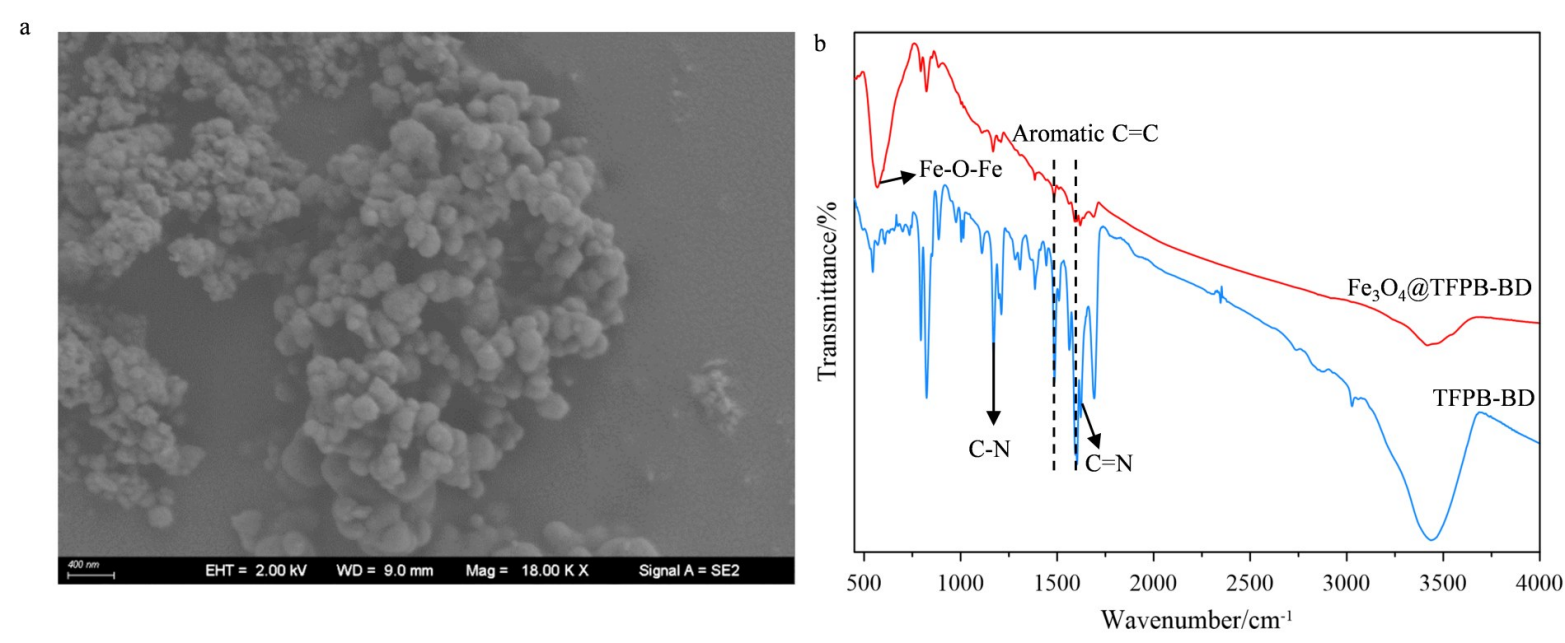
Fe_3_O_4_@TFPB-BD的（a）微观形貌图和（b）红外光谱图

### 2.2 磁固相萃取条件优化

MSPE过程基于溶质在溶液（流动相）与磁性吸附剂（固定相）之间的分配平衡原理。在MSPE教学实验中，引入“单因素变量设计法”，指导学生分别考察吸附剂用量、溶液pH、吸附时间及洗脱剂种类对萃取效率的影响。使学生掌握前处理条件与材料性能之间的内在关系，提升其实验设计与数据分析能力。

#### 2.2.1 吸附剂用量

学生在实验中通过对比不同用量（8~16 mg）Fe_3_O_4_@TFPB-BD对4种OUVFs的响应强度，直观认识到吸附剂用量对萃取效率的影响。结果如图5a所示，随着材料用量的增加，响应强度逐渐提高，并在14 mg处趋于平稳。因此，Fe_3_O_4_@TFPB-BD的用量设定为14 mg。此实验通过观察不同吸附剂用量下OUVFs的萃取效率变化，帮助学生理解吸附剂用量与有效吸附位点数量之间的关系。随着吸附剂用量的增加，材料提供的吸附位点增多，对OUVFs的萃取效率上升；当吸附剂用量增至一定水平后，吸附位点趋于饱和，继续增加用量，萃取效率也不再显著提高，从而引出了“吸附位点饱和”这一核心概念。

**图5:**
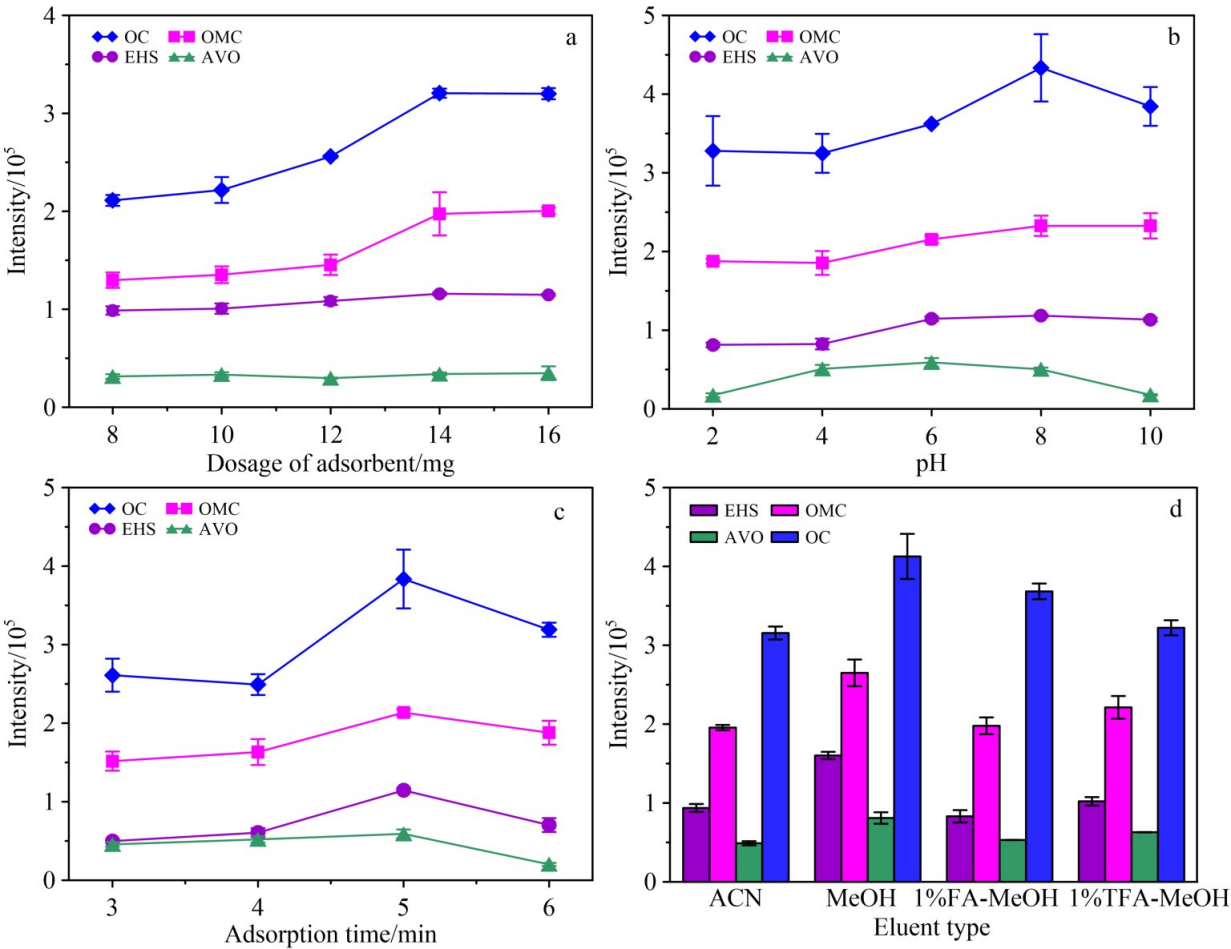
（a）吸附剂用量、（b）水样pH、（c）吸附时间和（d）洗脱剂种类对富集效率的影响（*n*=3）

#### 2.2.2 溶液的pH

鉴于OUVFs的分子结构中包含弱极性基团，其与Fe_3_O_4_@TFPB-BD之间的作用机制（如疏水作用、*π-π*堆积、氢键作用）易受溶液的pH影响^［17］^。因此，样品的pH设置为2.0~10.0进行富集实验。结果如图5b所示，在pH为8.0时4种OUVFs的响应强度最高，表明此时萃取效率最佳。而在强碱条件下，4种OUVFs的响应强度偏低，这可能与4种OUVFs的离子形态和Fe_3_O_4_@TFPB-BD的表面电荷有关。由表1可知，4种OUVFs的p*K*_a_为7.5~9.7，在强碱的样品溶液中它们呈现阴离子形态。而Fe_3_O_4_@TFPB-BD的等电点为4.4，在强碱条件下表面带有负电荷，与呈现阴离子形态的4种OUVFs之间存在静电斥力，导致萃取效率下降。通过该部分教学，学生可深入理解“吸附剂表面电荷状态-目标物存在形态-溶液pH环境”三者之间的协同关系，为实际样品分析中的条件设定提供理论依据。

#### 2.2.3 吸附时间

在考察不同吸附时间（3~6 min）的萃取效果后，学生发现多数目标物在5 min时即可达到吸附平衡（图5c）。这表明OUVFs已通过其与Fe_3_O_4_@TFPB-BD材料之间的相互作用，从水相转移并充分吸附到材料表面，此时可认为吸附达到平衡。因此，吸附时间确定为5 min。该实验帮助学生掌握动力学过程对吸附效率的影响，理解材料快速富集的重要性，并培养其在时间变量控制下的数据分析能力。

#### 2.2.4 洗脱剂的种类

学生通过比较采用不同溶剂（乙腈、甲醇、1%甲酸甲醇和1%三氟乙酸甲醇）洗脱时目标物的响应强度，最终确定甲醇为最优洗脱剂（图5d）。甲醇能够凭借其适当的极性和质子供给能力，能够通过竞争氢键、有效破坏OUVFs与Fe_3_O_4_@TFPB-BD之间的*π-π*共轭等关键作用力，从而实现OUVFs的高效解吸。该环节引导学生从分子相互作用层面理解洗脱过程的本质，进而关注洗脱剂极性、溶解能力、酸碱性等因素对解吸效率的影响。

### 2.3 方法学验证

在最佳的磁固相萃取条件下，采用不同加标水平（2、10、20、500、1 000、2 000 ng/L）的工作溶液进行前处理与UHPLC-MS/MS分析，以构建基质匹配标准曲线。以OUVFs的理论质量浓度（*x*， ng/L）为横坐标，其定量离子对的色谱峰面积（*y*）为纵坐标，通过最小二乘法进行线性回归，建立MSPE-UHPLC-MS/MS分析方法的标准曲线。结果如表2所示，4种OUVFs在2~2 000 ng/L范围内表现出良好的线性关系（相关系数>0.990 5），方法的检出限（LOD， *S/N*=3）及定量限（LOQ， *S/N*=10）分别为0.23~0.38 ng/L和0.77~1.24 ng/L。为评估所建立分析方法的重复性，选取低、中、高3个水平（20， 1 000和2 000 ng/L）进行加标试验，并计算相对标准偏差（RSD），所得到的日内精密度（*n*=6）为0.97%~14.89%，表明方法在痕量污染物分析中具有良好的稳定性。教学中除强调LOD、LOQ和RSD的定义与计算公式外，还特别引导学生思考方法在实际环境监测中的适用性与意义。尽管目前国内外标准中尚未对有机紫外防晒剂设定限值，但本研究建立的高灵敏度方法可为该类新污染物的环境行为研究、生态风险评估及未来标准制定提供可靠的数据支持，从而培养学生建立分析方法在实际应用中的有效性与价值认知。

**表2:** 4种OUVFs的分析方法性能参数

OUVF	Regression equation	Correlation coefficient	Linear range/（ng/L）	LOD/（ng/L）	LOQ/（ng/L）	RSD/%
OC	*y*=109.95*x+*1579.61	0.9955	2*‒*2000	0.38	1.24	7.89*‒*14.87
AVO	*y*=320.00*x*+67294.86	0.9998	2*‒*2000	0.29	0.94	9.15*‒*14.53
OMC	*y*=170.44*x‒*1876.29	0.9905	2*‒*2000	0.23	0.77	0.97*‒*12.44
EHS	*y*=11.65*x*+166.83	0.9973	2*‒*2000	0.34	1.13	9.48*‒*14.89

*y*： peak area； *x*： mass concentration， ng/L.

### 2.4 实际样品分析

在海水样品中检测出EHS（120.76 ng/L）和AVO（17.50 ng/L），其含量水平与文献报道数值相符^［6］^，证实海水是OUVFs暴露的潜在场景。同时为考察方法的准确性与适用性，本实验选取低、中、高（20、1 000和2 000 ng/L）3个水平对海水样品进行加标回收率测试。结果显示，4种OUVFs的加标回收率为87.54%~120.00%，RSD为3.77%~13.48%，表明该方法具有实用性。图6展示了海水样品中4种OUVFs加标前后的色谱图。此部分实验强化了学生对“复杂基质样品处理-定量分析-污染暴露评估”的整体认知，提升其将分析结果用于真实环境分析的能力。

**图6:**
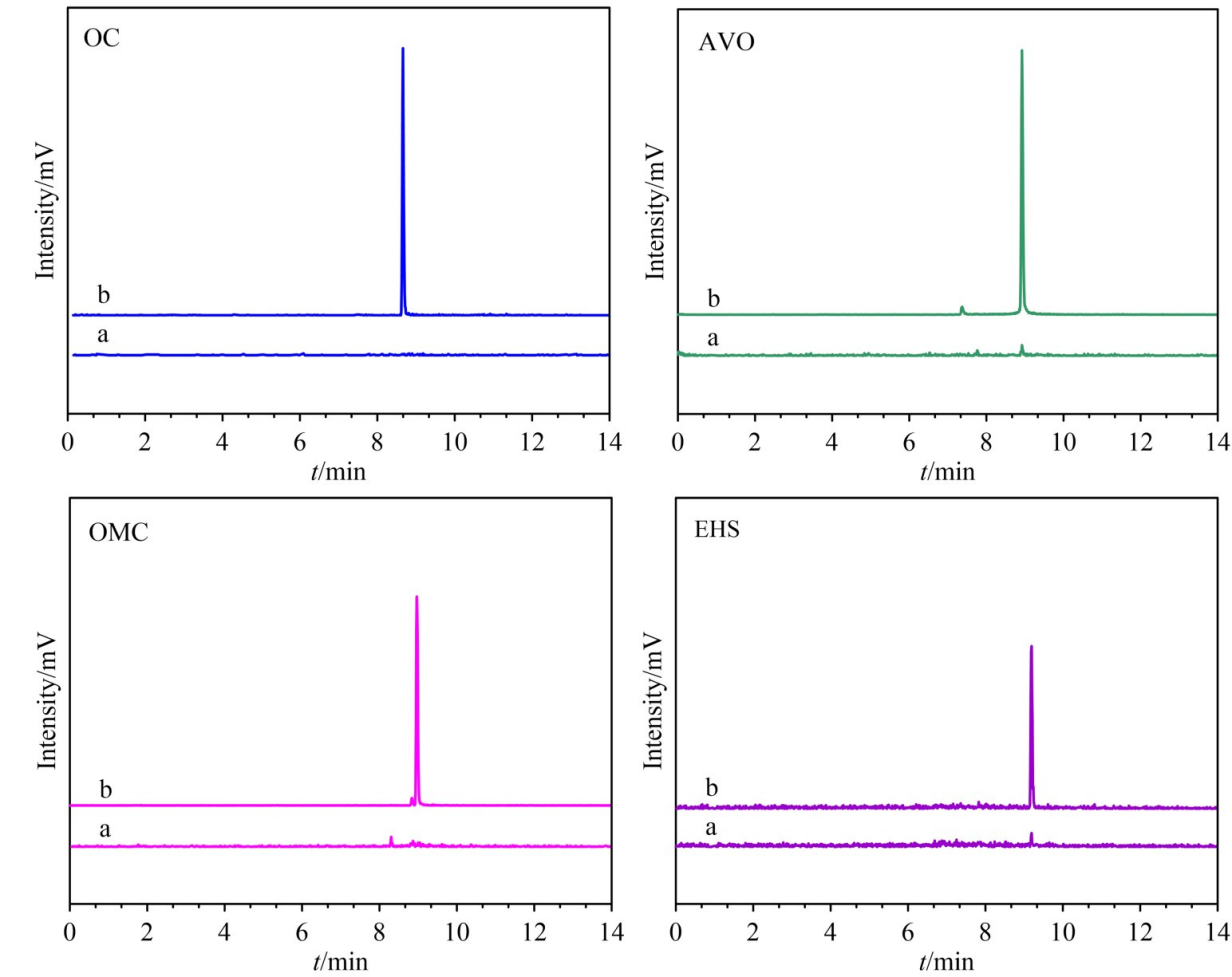
（a）实际海水样品和（b）加标（2 000 ng/L）海水样品中4种OUVFs的色谱图

## 3 实验的组织实施及教学反思

### 3.1 实验的组织实施

本实验共设置16学时，分两次课完成：第一次课（8学时）进行磁固相萃取条件优化和仪器检测，第二次课（8学时）开展方法学验证和实际样品分析。课程以“功能材料构建-样品前处理-痕量污染物分析”为主线，结合新污染物背景与大型仪器应用需求，面向环境类本科生开设综合实验教学模块。课程设计遵循“以学生为中心”的教育理念，围绕“自主预习、项目驱动、教师引导、实验反思”的教学路径组织实施，致力于培养学生综合运用材料科学与分析化学知识解决实际环境问题的能力。

课前，教师通过构建教学资源包提供导学材料，包括新污染物前沿背景资料、MCOF材料合成与表征过程、MSPE技术原理以及UHPLC-MS/MS的基本操作视频，帮助学生提前形成知识框架。学生需在课前完成自主预习任务，了解实验目标、掌握基本步骤，并结合实际污染物检测案例提出预设问题。为促进学生思考与投入，课前教师还布置引导性任务，如绘制实验流程图、查阅目标物物理化学性质等，帮助其构建实验方案的基础认知。

进入实验阶段，学生在教师启发下4人一组开展实验操作。整个过程中，教师重点引导学生围绕实验设计的合理性与变量设置展开讨论，鼓励其提出问题并结合实验现象进行解释。例如，在进行吸附剂用量、pH和吸附时间的优化实验中，教师将引导学生思考材料孔结构、官能团与目标物之间的作用机制，从而建立起参数设置与材料性能之间的关联理解。在仪器分析环节，学生需独立完成标准曲线绘制、加标回收率计算和方法学指标评价，逐步掌握从信号读取到定量分析的完整逻辑链条。

实验完成后，学生需在课后整理实验数据并撰写完整报告，不仅涵盖实验过程与结果分析，还需进行误差分析和团队协作总结。教师则需对学生实验记录、方法设计与数据处理能力进行综合评价，并在教学总结中识别学生在认知与技能层面的薄弱点，反馈至后续教学方案优化之中。

### 3.2 教学反思

本研究通过将共价有机骨架材料的合成与表征、磁性固相萃取技术以及超高效液相色谱-质谱联用方法开发有机整合，体现了材料化学、分析化学与环境工程等多学科的深度交叉。该综合性实验旨在突破学科界限，引导学生融合《有机化学》《环境监测》《分析化学》及《仪器分析》等多门课程知识，以应对环境问题固有的复杂性与综合性。通过这一完整链条的实践，学生能够将分散的理论知识与实验技能系统串联，形成整体性解决方案，切实锻炼跨学科思维与综合解决复杂环境问题的能力。

建议该实验在课程体系中以模块化形式嵌入“样品前处理技术”“现代环境分析方法”等核心课程，灵活融入不同教学阶段。对部分高校或学生仪器资源有限的情况，可引入虚拟仿真实验结合真实操作演示的模式，结合材料三维结构模拟、色谱分离流程动画及谱图解析交互软件，形成“实训、虚拟、案例”的混合教学方案，提升教学可复制性与推广价值。此外，在教学过程中应注重将课程思政元素有机融入知识传授与技能培养之中。通过引导学生思考当前环境监测中“卡脖子”技术问题与国产仪器的发展现状，使学生认识到高端分析仪器自主研发的战略意义。例如，在液相色谱-串联质谱分析环节，教师可引导学生关注国产UHPLC-MS/MS系统的技术突破，强化学生科技报国的价值认同。通过讲解新污染物纳入国家监管名单的政策背景，引发学生对环境健康风险的社会责任意识，逐步培育其绿色发展理念与生态文明素养。

## 4 结语

本研究基于“功能材料制备-前处理优化-痕量分析”的实验框架，以真实环境样品中OUVFs的富集分析为切入点，创新性地将共价有机骨架材料的制备表征、磁固相萃取技术和超高效液相色谱-质谱联用技术有机整合，构建了面向环境类本科生的综合实验教学体系。通过本实验的系统训练，学生不仅掌握了材料合成与表征、样品前处理参数优化以及大型仪器操作与数据分析等核心技能，更在解决真实环境问题的过程中，培养了设计研究方案、评估方法效能及解析污染特征的综合创新能力。在教学实践中，通过课前自主学习、课中实验操作和课后反思总结，实现了理论与实践的深度融合。教学过程中，本实验将国家生态环境治理需求、国产分析仪器发展现状等思政元素自然融入教学环节，使学生在掌握专业技能的同时，深化了对环境分析工作社会价值的认识。这种融合学科前沿与基础技能培养的教学模式，为环境分析类实验课程的改革创新提供了有益借鉴，对培养具备多学科交叉能力的复合型环境人才具有重要意义。未来将通过开发虚拟仿真预习系统，优化资源配置，实现课程深度与广度的双向拓展。
